# How the Infestation Level of *Varroa destructor* Affects the Distribution Pattern of Multi-Infested Cells in Worker Brood of *Apis mellifera*

**DOI:** 10.3390/vetsci7030136

**Published:** 2020-09-17

**Authors:** Ignazio Floris, Michelina Pusceddu, Alberto Satta

**Affiliations:** Department of Agricultural Science, University of Sassari, Viale Italia 39, 07100 Sassari, Italy; mpusceddu@uniss.it (M.P.); albsatta@uniss.it (A.S.)

**Keywords:** mite, reproductive rate, worker brood, infestation level, longevity, distribution, model, *Apis mellifera*

## Abstract

The mite *Varroa destructor*, the main ectoparasite of honey bees, is a threat to apiculture worldwide. Understanding the ecological interactions between *Varroa* and honeybees is fundamental for reducing mite impact in apiaries. This work assesses bee colonies with various *Varroa* infestation levels in apiaries to determine: (1) the relationship between multi-infested brood cells and brood infestation level, (2) the damage caused by *Varroa* to parasitized honey bee pupae, and (3) mite reproduction rate at various infestation levels. Data were collected from 19 worker brood combs, each from a different colony, ranging from 160 to 1725 (mean = 706) sealed cells per comb. Mite distribution was aggregated, ranging from about 2% to 74% infested cells per comb. The percentage of cells invaded by one, two, three, four, or more than four foundress mites, as a function of infestation level, was estimated by five highly significant (*p* < 0.0001) second-degree polynomial regression equations. The correction factors found could increase the precision of prediction models. *Varroa* fertility and adult bee longevity decreased as multi-infestation levels increased, and the implications of this relationship are discussed. Finally, these findings could improve sampling methods and the timing of mite treatments in apiaries, thus favoring sustainable management strategies.

## 1. Introduction

The parasitic mite *Varroa destructor* was originally confined to the Eastern honey bee *Apis cerana* [1]. After a shift to a new host, *A. mellifera*, and a worldwide dispersion, this mite has become the most serious threat to honeybees [1,2]. *Varroa* has had a fundamental role in the decline of honeybee colonies observed all over the Northern Hemisphere in the last few decades [3,4].

Many factors of the host and the parasite affect the population growth of *Varroa* in honeybee colonies [5,6], such as the worker brood availability. In fact, the number of brood cells and/or the fertility of the mites and population growth are significantly correlated [7,8,9]. Therefore, it is likely that the population dynamic of the bee colony significantly influences the development of *Varroa* infestation. It is known that the average number of adult female offspring produced by a single mother mite invading a worker brood cell ranges between 1.2–1.5, whereas this reproduction rate rises to 2.2–2.6 in drone brood cells due to their longer capping period [10,11,12]. Moreover, in multiple-infested brood cells, the reproductive rate per female mite is significantly reduced [12,13,14,15]. Consequently, the number of brood cells throughout the season, the temporal pattern of brood availability, and the percentage of drone brood, among other factors [1], can have an impact on the reproduction of the *Varroa* population. This is described in some population dynamic models of *Varroa* mites and honeybees [12,16,17]. Furthermore, in the honey bee brood, it is not uncommon to find sealed cells with bee larvae infested by two, three, or more female mites, while many other cells remain uninfested. This behavior suggests that the distribution of the mite among brood cells is not random but, rather, aggregated [18,19,20,21]. However, it should also be pointed out that some other studies do not support the aggregation hypothesis [12,22]. Differences in the statistical approach and data collection methods adopted probably led to different interpretations in these studies. Despite that, what clearly emerges statistically is that the mean values of brood infestation are always lower than the respective variances, which is a basic assumption to demonstrate the tendency to aggregation from an ecological point of view [23,24].

An aspect that remains to be explained is the different attractiveness of the brood cells, which is at the basis of the aggregation behavior, such as possible chemical sources of attractiveness [2]. Aggregation could favor exogamy and may have an adaptive value for *Varroa*, but it is unknown whether this phenomenon is related to an aggregation pheromone, to the higher attractiveness of certain bee larvae, or to other biotic and abiotic factors [2].

One of the main sources of error in the application of sampling techniques is the aggregate distribution, especially when cutting honeycomb parts [25,26]. In fact, the variability of infestation between different areas of the same comb or between different combs [27,28], due to the irregular distribution of *Varroa*, may lead to substantial differences in sampling results. An accurate estimation of brood infestation, based on precise knowledge of the mite spatial distribution pattern and its interpretation by specific models (e.g., Iwao’s regression method [20]), favors the development adoption of appropriate sampling plans (e.g., stratified random sampling, cross sampling or, for practical purposes, sequential sampling) [19,20,26]. Better understanding the basic ecology of the mite is useful for several other reasons: (1) the correct estimation of the infestation level of the brood can help to determine the most appropriate timing of treatments used for *Varroa* control, as done in the sustainable integrated pest management (IPM); (2) the damage caused by *Varroa* to larvae or pupae depends on the infestation level and the number of mites entering the cell, as demonstrated by the correlations between pathogen loads (positive) or colony strength (negative) and *Varroa* infestation rate [29,30]; thus, knowing the mite distribution pattern can be useful in determining the extent of brood damage as a function of infestation level; (3) an accurate estimate of the percentage of cells with a specific number of female mites allows us to correctly assess the *Varroa* reproduction rate because the increased competition among the offspring mites for food and space in a multi-infested cell can decrease mite fertility. In fact, in multiple-invaded drone and worker brood cells, the reproductive rate per female mite is significantly reduced [12,14,15,31].

The available predictive models of mite dynamics [11,12,16,17,32] do not consider the effects of multi-infested brood cells on the development of *Varroa* infestation, thus associating mite growth rates simply with brood availability (distinguishing only between male and female brood), or they refer to a Poisson distribution, which is not accurate enough to represent the real behavior of *Varroa* in bee brood [33]. Some studies have shown an aggregated distribution of *Varroa* in the brood [19,20], associated with increases in multi-infestation as population density grows, with consequent effects on mite development and reproduction rate, as well as on the extent of damage to bee colonies. It is important to highlight that in environmental conditions favorable for the constant presence of brood in the hives throughout seasons, such as in the Mediterranean area, it is crucial to correctly estimate the percentage of cells infested by one or more mites. This allows for the definition of more realistic simulation models of the development dynamic of *Varroa*, in line with its statistical spatial distribution, thus promoting more sustainable and efficient mite control.

This work assesses bee colonies with various levels of natural infestations by *Varroa* in apiaries to determine: (1) the relationship between multi-infested brood cells and brood infestation level, (2) the damage caused by *Varroa* to parasitized honey bee pupae in terms of bee longevity, and (3) the effect of infestation level on mite reproduction rate. Based on data collected from numerous worker brood combs with percentages of infested cells ranging from about 2% to 74%, five second-degree polynomial regression equations were developed to estimate the percentage of cells invaded by one, two, three, four, and more than four foundress mites according to the infestation level. The work also discusses the implications of these relationships on the reduction of *Varroa* fertility and longevity of adult bees. Our findings could favor sustainable management strategies by improving predictive models, sampling methods, and timing of mite treatments in apiaries.

## 2. Materials and Methods

The work was carried out in the experimental apiary of the University of Sassari located in Ottava (40°46′23″ N; 8°29′34″ E), Province of Sassari, Italy, from late summer to early fall (September–October) in 2018. The apiary was composed of *A. mellifera* colonies kept in Dadant–Blatt standard hives naturally infested by *Varroa*, at various infestation levels.

In total, 19 combs containing worker sealed brood were taken from the central position of the nest of 19 hives, 3 days after cell sealing started, and maintained at −20 °C until inspection. For each honeycomb, all sealed brood cells were inspected, and the number of foundress mites present in each infested cell was recorded. The number of sealed brood cells in each comb ranged from 160 (comb no. 18) to 1725 (comb no. 2), with an average value of 706 (Table 1).

The following descriptive variables were calculated for each comb: (a) the total number of sealed brood cells, (b) the total number of female (foundress) mites, (c) the average number of female mites per brood cell, and (d) the number and percentage of cells containing one, two, three, four, and more than four foundress mites per cell. These data were used to derive second-degree polynomial regression equations to correlate the average number of foundress mites per infested cell with the percentage of cells containing one, two, three, four, or more than four foundress mites per cells, separately.

The regression equations obtained were used to derive two other second-degree regression equations, useful to describe the mean reproduction rate (fecundity) of female mites and the percentage of longevity reduction of adult bees parasitized by mites in the preimaginal stage as a function of the mean number of mother mites per infested cell. In order to develop these two equations, we simulated 17 different distributions of foundress mites, considering a constant number of cells available for invasion (1000) and an increasing number of mites (from 10 to 2500). After that, the average fecundity of foundress mites for each mite distribution was calculated considering the following average values of mite offspring: 1.45, 1.32, 1.25, 0.87, and 0 mites in cells with one, two, three, four, and more than four foundress mites, respectively [12]. To calculate the average longevity of workers that have emerged from the infested cells, the following percentage reduction in lifespan was considered: 2%, 10%, 20%, 40%, and 80% in cells with one, two, three, four, and more than four foundress mites, respectively [16].

## 3. Results

**Figure 1:**
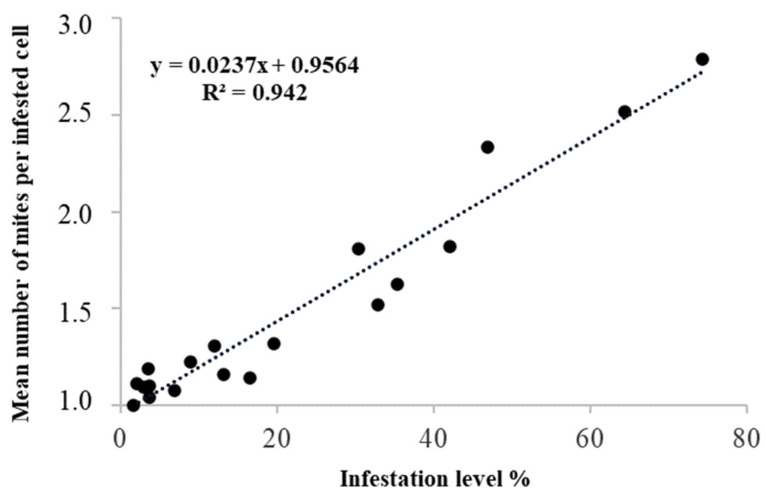
Relationship between the percentage of *Varroa* infestation level and the mean number of foundress mites per infested cells of worker brood.

The number of sealed brood cells, infested brood cells, and foundress mites in each of the 19 inspected combs, the percentage of infestation per comb, and the mean number of female mites per infested cell are given in Table 1. The infestation level of *Varroa* ranged from 1.7% to 74.3% in the inspected combs, and the mean number of foundress mites per infested cell varied from 1 to 2.8 (Table 1). Between these last two variables, a highly significant, linear and positive, relationship (Df = 1; F = 277.4; *p* < 0.00001) was observed (Figure 1), with approximately 94% of the variability of the mean number of foundress mites per infested cell explained by the infestation level (R^2^ = 0.942). This relationship clearly shows that as the level of infestation increases, the phenomenon of multi-infestation increases.

**Figure 2:**
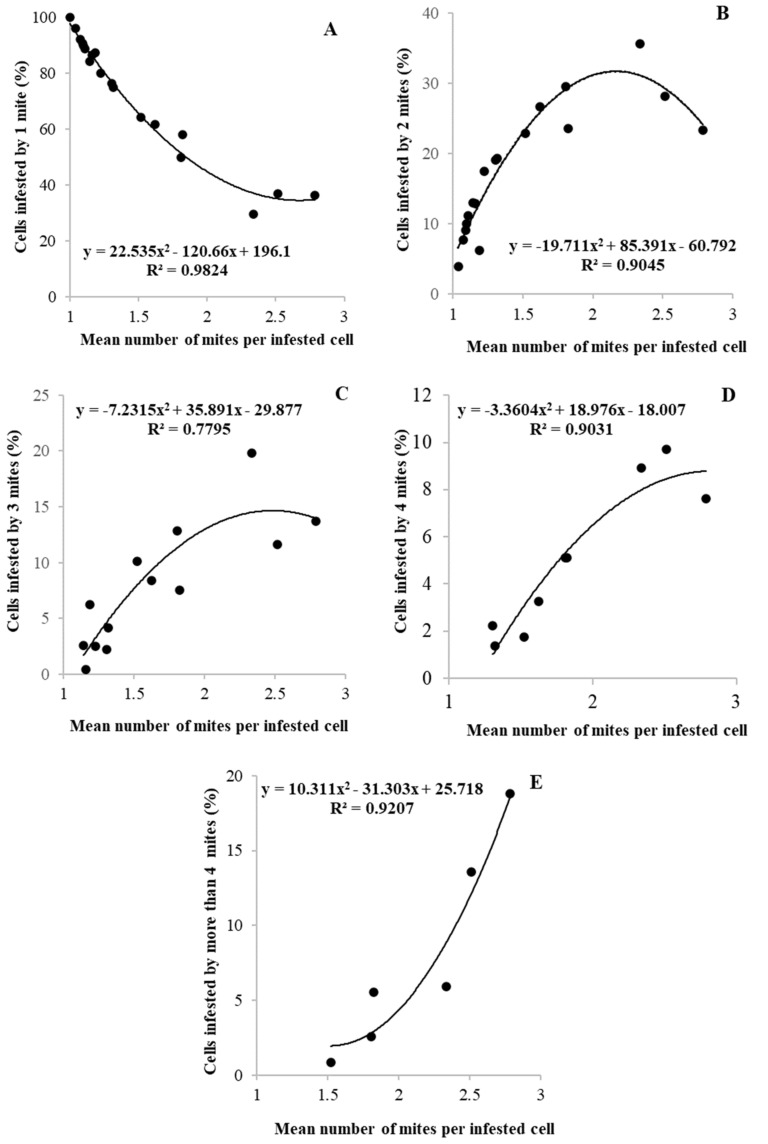
Relationship between the mean number of foundress mites per infested worker brood cells and the percentage of cells invaded by one (**A**), two (**B**), three (**C**), four (**D**), and more than four (**E**) foundress mite. In each graph, each dot represents a colony.

From the raw data, we calculated, for each infestation level, the percentage of cells with one, two, three, four, and more than four foundress mites. Then, we correlated these parameters with the mean number of female mites per infested cells. The equations that better fitted the relationship between these variables were second-degree polynomials, as shown in Figure 2.

The second-degree polynomial curves (Figure 2A–E), all highly significant (*p* < 0.0001), allow us to evaluate the distribution of the foundress mites inside the brood cells for different infestation levels. In fact, we can observe that when the level of infestation increases, the progressive decrease in the percentage of cells containing only one mother mite (R^2^ = 0.9824; Figure 2A) is compensated by the growth of cells containing two (R^2^ = 0.9045; Figure 2B), three (R^2^ = 0.7795; Figure 2C), four (R^2^ = 0.9031; Figure 2D) or more than four (R^2^ = 0.9207; Figure 2E) foundress mites. Consequently, once the number of mites passing from the phoretic to the reproductive phase is established and once the number of cells available to be invaded by the mite is known, e.g., by using a predictive model [17,34], it could be possible to define the distribution of female mites in brood cells for each level of infestation.

**Figure 3:**
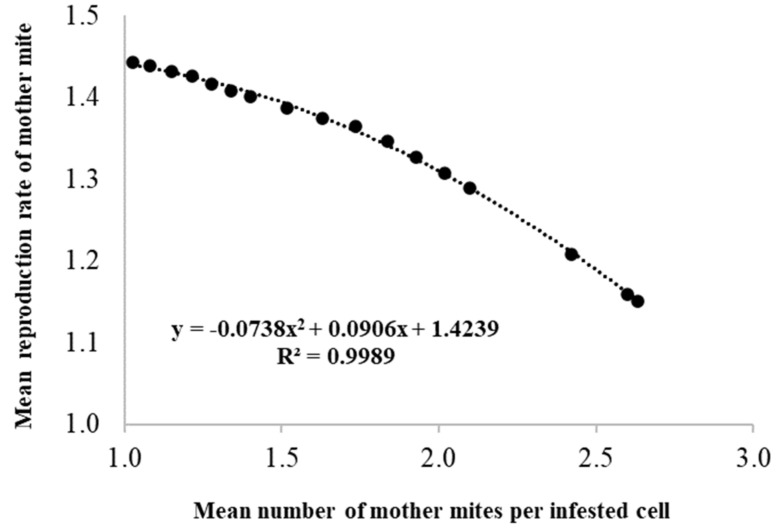
Relationship between the mean number of foundress mites per infested cells of worker brood and the reduction of their reproduction rate.

**Figure 4:**
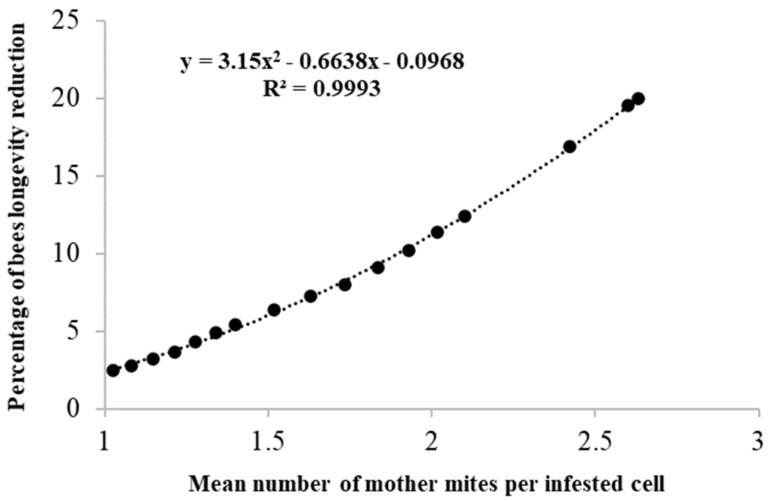
Relationship between the mean number of foundress mites per infested cells of worker brood and the longevity reduction of honeybee adults.

Starting from the studies of Martin [12] and DeGrandi-Hoffmann and Curry [16], which showed the effects of infestation on mite reproduction rate and bee longevity, we can highlight the additional effects of multi-infestation. Therefore, considering that the *Varroa* reproduction rate decreases with increasing multi-infestation, if the distribution of multi-infested cells is known for each infestation level, we can also calculate the average reproduction rate of *Varroa*. For this purpose, by simulating a series of mite distributions at levels of growing infestation (*n* = 17), a regression curve was derived to express the negative relationship between the average fecundity per female mite and the average number of foundress mites for infested cell (R^2^ = 0.9989; Figure 3). These data could also be used to improve the models on the *Varroa* dynamic. The same criterion used above was applied to derive a regression curve describing the decrease in the longevity of adult bees as a function of the average number of mites per infested cell (R^2^ = 0.9993; Figure 4). In this case, the information obtained could be used to better understand the effects of the increasing infestation levels on the bee population dynamic.

## 4. Discussion

Based on the data collected under our specific experimental conditions, our findings suggest applications of practical relevance in terms of improving *Varroa* control strategies. Differences due to seasonal or environmental variations are possible but likely limited to the range of infestation levels detected. In fact, these differences should not affect the relationship between infestation and multi-infestation levels nor the effects of multi-infestation because the latter is proven to be density-dependent [13]. This is clearly evidenced by the observed increase in multi-infestation as the infestation level increases. Furthermore, based on the model of De Grandi-Hoffmann and Curry [16], control treatments applied against *Varroa* in late summer provide the best chances for the survival of heavily infested colonies. Therefore, surveys conducted in late summer, as in our case, are particularly important in Mediterranean environments. However, it should also be considered that colony survival thresholds for mite populations and the effectiveness of miticides are dependent on the climate and the yearly brood dynamic.

The analysis of the data collected in our study revealed, according to the principles established by ecological methods [23,24], an aggregate distribution of *Varroa* in the brood. Such distribution can be interpreted by different models, such as Iwao’s regression method, the negative binomial, and other interpretative models [19,20]. The aggregate distribution of the mite in the brood is associated with multi-infestation, with mites tending to concentrate in some areas of the brood comb, thus increasing the possibility that some of them may invade the same cell. However, the factors causing mite aggregation are still unknown [2].

Among the possible applications of our findings, the definition of more precise prediction models for the development of *Varroa* infestation is one of the most interesting aspects. In fact, based on our findings, we can provide significant correction factors, previously unknown in the literature, to define the evolution of *Varroa* infestation, better representing the behavior of the mite in apiary conditions. Differently from previous studies, in our work, all the cells of the combs were examined according to their natural distribution, without preselection of combs or honeycomb areas with cells of the same age and so on, and the observations were not conducted under laboratory conditions. Therefore, our data reflect the natural behavior of the mite in honeybee colonies in their environment.

Our findings also have an important practical and scientific impact on the definition of more appropriate and precise sampling methods. In particular, the detected aggregated mite distribution suggests, from a statistical point of view, the need for a stratified sampling for a correct evaluation of brood infestation level. For practical purposes, sequential sampling [19,20], based on predefined infestation thresholds, is appropriate, whereas for experimental designs, crossed sampling [35] is recommended.

Finally, knowing the levels of *Varroa* infestation in apiaries and understanding the effects of their presence on beehives allows us to make correct decisions on treatment timing. This is particularly important in areas where bee brood are constantly present in the hives during the year, as in the case of Mediterranean environments, where chemical treatments are still the main means to control *Varroa* mite infestations, with the aim of limiting undesirable toxicological and pathological side effects of the applied products and of the mite infestation, respectively.

## Figures and Tables

**Table 1:** The number of sealed and infested brood cells and foundress mites obtained by inspecting 19 combs sampled from 19 different colonies of honey bees naturally infested by *Varroa* in the apiary. The percentage of infestation and the mean number of foundress mites per infested cell were calculated for each comb.

Comb	Number of Sealed Brood Cells	Number of Infested Cells	Number of Foundress Mites	Percentage of Infestation ^a^	Mean Number of Foundress Mites Per Infested Cell
1	483	8	8	1.7	1.0
2	1725	36	40	2.1	1.1
3	738	22	24	3.0	1.1
4	442	16	19	3.6	1.2
5	545	20	22	3.7	1.1
6	1378	51	53	3.7	1.0
7	374	26	28	7.0	1.1
8	445	40	49	9.0	1.2
9	738	89	116	12.1	1.3
10	1652	218	253	13.2	1.2
11	466	77	88	16.5	1.1
12	740	145	191	19.6	1.3
13	256	78	141	30.5	1.8
14	691	227	345	32.9	1.5
15	1040	368	597	35.4	1.6
16	1066	449	818	42.1	1.8
17	215	101	236	47.0	2.3
18	160	103	259	64.4	2.5
19	265	197	549	74.3	2.8

**^a^** Data are listed in ascending order according to the percentage of infestation.
