# Correction: Floris, I., et al. How the Infestation Level of *Varroa destructor* Affects the Distribution Pattern of Multi-Infested Cells in Worker Brood of *Apis mellifera*. *Veterinary Science* 2020, *7*, 136

**DOI:** 10.3390/vetsci7040202

**Published:** 2020-12-14

**Authors:** Ignazio Floris, Michelina Pusceddu, Alberto Satta

**Affiliations:** Department of Agricultural Science, University of Sassari, Viale Italia 39, 07100 Sassari, Italy; mpusceddu@uniss.it (M.P.); albsatta@uniss.it (A.S.)

We have recently been made aware by the reviewer and the Journal Editorial Offices of the following weaknesses in our recent paper [1]:

## 1. Introduction

Introduction, second paragraph, line 14 currently reads as follows:

[This behavior suggests]

We would like to make the following corrections:

[This suggests]

Introduction, fifth paragraph, line 17 currently reads as follows:

[It is important to highlight that in environmental conditions favorable for the constant presence of brood in the hives throughout seasons, such as in the Mediterranean area, it is crucial to correctly estimate the percentage of cells infested by one or more mites.]

Add at the end of the sentence the reference [34].

[It is important to highlight that in environmental conditions favorable for the constant presence of brood in the hives throughout seasons, such as in the Mediterranean area, it is crucial to correctly estimate the percentage of cells infested by one or more mites [34]]

## 2. Materials and Methods

At the end of the section Materials and Methods add the sentence:

[Data availability: The complete datasets analyzed during the current study are available from the corresponding author on reasonable request.]

## 3. Results

Starting from the line 19 of the results section remove the two following consecutive sentences:

[Consequently, once the number of mites passing from the phoretic to the reproductive phase is established and once the number of cells available to be invaded by the mite is known, e.g., by using a predictive model [17,34], it could be possible to define the distribution of female mites in brood cells for each level of infestation.]

[Starting from the studies of Martin [12] and DeGrandi-Hoffmann and Curry [16], which showed the effects of infestation on mite reproduction rate and bee longevity, we can highlight the additional effects of multi-infestation.]

At the beginning of the next sentence, replace:

[Therefore]

with

[Considering]

Results line 31: Remove the sentence:

[These data could also be used to improve the models on the Varroa dynamic]

## 4. Discussion

Discussion, first paragraph, line 7:

Add the following sentence and add a new reference 35 [2]:

[This phenomenon also has important effects on the genetic structure of the population of Varroa. In fact, as observed by Beaurepaire et al. [35], the increase in the co-infestation rate of brood cells also coincides with an increase in the recombined lines in the mite populations. Therefore, if treatments against Varroa are carried out before the recombination phase has taken place, the inbreeding will greatly promote the fixation of the alleles for acaricide resistance [35]].

Discussion, third paragraph, line 2

Replace the sentence:

[In fact, based on our findings, we can provide significant correction factors, previously unknown in the literature, to define the evolution of Varroa infestation, better representing the behavior of the mite in apiary conditions]

with:

[In fact, based on our second-degree polynomial curves it could be possible to define the distribution of female mites in brood cells and their rate of reproduction for each level of infestation. Therefore, these equations provide significant correction factors, previously unknown in the literature, to define the evolution of Varroa infestation, better representing the behavior of the mite in apiary conditions]

Discussion, third paragraph, line 5 add the follow sentence

[In addition, the information obtained in the reduction of adult bee longevity as a function of the average number of mites per infested cell, could be used to better understand the effects of the increasing infestation levels on bee population dynamic.]

Discussion, fifth paragraph, line 5:

The indication of reference 35 has to be change in reference 36.

## 5. References

Replace reference 4:

[Neumann, P.; Carreck, L. Honey bee colony losses. *J. Apic. Res*. **2010**, 49, *1–6*.]

with

[Neumann, P.; Carreck, N.L. Honey bee colony losses. *J. Apic. Res*. **2010**, 49, *1–6*.]

Replace reference 6:

[Fries, I.; Camazine, S.; Sneyd, J. Population dynamics of *Varroa jacobsoni*: A model and a review. *Bee World* **1994**, *75*, 4–28.]

with

[Fries, I.; Camazine, S.; Sneyd, J. Population Dynamics of *Varroa jacobsoni*: A Model and a Review. *Bee World* **1994**, *75*, 5–28.]

Replace reference 9:

[Lodesani, M.; Crailsheim, C.; Moritz, R.F.A. Effect of some characters on the population growth of mite *Varroa jacobsoni* in *Apis mellifera* L. colonies and results of a bi-directional selection. *J. Appl. Entomol*. **2002**, *126*, 130–137.]

with

[Lodesani, M.; Crailsheim, K.; Moritz, R.F.A. Effect of some characters on the population growth of mite *Varroa jacobsoni* in *Apis mellifera* L. colonies and results of a bi-directional selection. *J. Appl. Entomol*. **2002**, *126*, 130–137.]

Replace reference 12:

[Martin, S.J. Reproduction of *Varroa jacobsoni* in cells of *Apis mellifera* containing one or more mother mites and the distribution of these cells. *J. Apicult. Res*. **1995**, *34*, 187–196.]

with

[Martin, S.J. Reproduction of *Varroa jacobsoni in* cells *of Apis mellifera* containing one or more mother mites and the distribution of these cells. *J. Apic. Res*. **1995**, *34*, 187–196.]

Replace reference 16:

[DeGrandi-Hoffman, G.; Curry, R. A mathematical model of *Varroa* mite (*Varroa destructor* Anderson and Trueman) and honeybee (*Apis mellifera* L.) population dynamics. *Internat. J. Acarol*. **2004**, *30*, 259–274.]

with

[DeGrandi-Hoffman, G.; Curry, R. A mathematical model of *Varroa* mite (*Varroa destructor* Anderson and Trueman) and honeybee (*Apis mellifera* L.) population dynamics. *Int. J. Acarol*. **2004**, *30*, 259–274.]

Replace reference 22:

[Salvy, M., Capowiez, Y., Le Conte, Y.; Clément, J.-L. Does the spatial distribution of the parasitic mite Varroa jacobsoni Oud. (Mesostigmata: Varroidae) in worker brood of honey bee Apis mellifera L. (Hymenoptera: Apidae) rely on an aggregative process? *Naturwissenschaften* **1999**, 86, *540–543*.]

with

[Salvy, M., Capowiez, Y., Le Conte, Y.; Clément, J.L. Does the spatial distribution of the parasitic mite varroa jacobsoni Oud. (Mesostigmata: varroidae) in worker brood of honey bee apis mellifera L. (Hymenoptera: apidae) rely on an aggregative process? *Naturwissenschaften* **1999**, 86, *540–543*.]

Replace reference 26:

[Dietmann, V.; Nazzi, F.; Martin, S.J.; Anderson, D.L.; Locke, B.; Delaplane, K.S.; Wauquiez, Q.; Tannahill, C.; Frey, E.; Ziegelmann, B.; et al. Standard methods for varroa research. *J. Apic. Res*. **2013**, 52, *1–54*.]

with

[Dietemann, V.; Nazzi, F.; Martin, S.J.; Anderson, D.L.; Locke, B.; Delaplane, K.S.; Wauquiez, Q.; Tannahill, C.; Frey, E.; Ziegelmann, B.; et al. Standard methods for varroa research. *J. Apic. Res.* **2015**, 52, *1–54*.]

Replace reference 29:

[Natsopoulou, M.N.; McMahon, D.P.; Doublet, V.; Frey, E.; Rosenkranz, P.; Paxton, R.J. The virulent, emerging genotype B of *Deformed wing virus* is closely linked to overwinter honeybee worker loss. *Sci. Rep.* **2017**, *7*, 5242.]

with

[Natsopoulou, M.E.; McMahon, D.P.; Doublet, V.; Frey, E.; Rosenkranz, P.; Paxton, R.J. The virulent, emerging genotype B of *Deformed wing virus* is closely linked to overwinter honeybee worker loss. *Sci. Rep.* **2017**, *7*, 5242.]

Replace reference 34:

[Boot, W.J.; Sisselaar, D.J.A.; Calis, J.N.M.; Beetsma, J. Factors affecting invasion of *Varroa jacobsoni* (Acari: Varroaidae) into honey bee, *Apis mellifera* (Hymenoptera: Apidae), brood cells. *B. Entomol. Res.* **1994**, *84*, 3–10.]

with

[Boot, W.J.; Sisselaar, D.J.A.; Calis, J.N.M.; Beetsma, J. Factors affecting invasion of *Varroa jacobsoni* (Acari: Varroaidae) into honey bee, *Apis mellifera* (Hymenoptera: Apidae), brood cells. *Bull. Entomol. Res.* **1994**, *84*, 3–10.]

The authors would like to apologize for any inconvenience caused to the readers by these changes.
